# From Nature to Lab: Sustainable Bacterial Cellulose Production and Modification with Synthetic Biology

**DOI:** 10.3390/polym15163466

**Published:** 2023-08-18

**Authors:** Vid Potočnik, Selestina Gorgieva, Janja Trček

**Affiliations:** 1 Department of Biology, Faculty of Natural Sciences and Mathematics, University of Maribor, 2000 Maribor, Slovenia; vid.potocnik.2@gmail.com; 2 Faculty of Mechanical Engineering, Institute of Engineering Materials and Design, University of Maribor, 2000 Maribor, Slovenia; selestina.gorgieva@um.si; 3 Faculty of Chemistry and Chemical Engineering, University of Maribor, 2000 Maribor, Slovenia

**Keywords:** acetic acid bacteria, *Komagataeibacter*, *Novacetimonas*, bacterial cellulose, sustainable production, agricultural waste, food waste, genetic engineering, synthetic biology, biomaterial

## Abstract

Bacterial cellulose (BC) is a macromolecule with versatile applications in medicine, pharmacy, biotechnology, cosmetology, food and food packaging, ecology, and electronics. Although many bacteria synthesize BC, the most efficient BC producers are certain species of the genera *Komagataeibacter* and *Novacetimonas*. These are also food-grade bacteria, simplifying their utilization at industrial facilities. The basic principles of BC synthesis are known from studies of *Komagataeibacter xylinus*, which became a model species for studying BC at genetic and molecular levels. Cellulose can also be of plant origin, but BC surpasses its purity. Moreover, the laboratory production of BC enables in situ modification into functionalized material with incorporated molecules during its synthesis. The possibility of growing *Komagataeibacter* and *Novacetimonas* species on various organic substrates and agricultural and food waste compounds also follows the green and sustainable economy principles. Further intervention into BC synthesis was enabled by genetic engineering tools, subsequently directing it into the field of synthetic biology. This review paper presents the development of the fascinating field of BC synthesis at the molecular level, seeking sustainable ways for its production and its applications towards genetic modifications of bacterial strains for producing novel types of living biomaterials using the flexible metabolic machinery of bacteria.

## 1. Review Contents

**Scheme 1:**
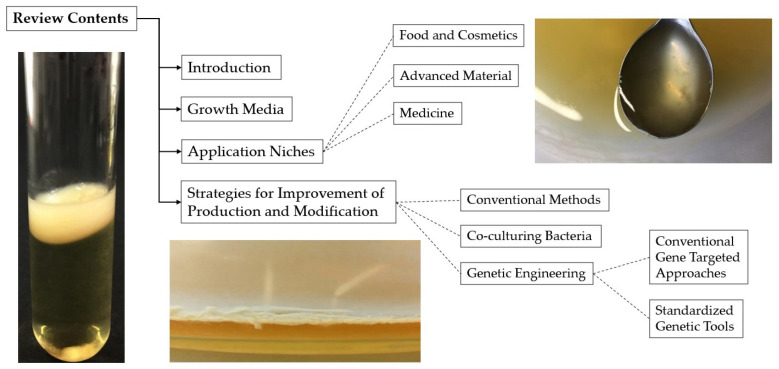
An overview of the sections covered in this review.

The Scheme 1 gives an overview of the sections covered in this review.

## 2. Bacterial Cellulose as Featured Nanomaterial

### 2.1. Introduction to Bacterial Cellulose

**Figure 1:**
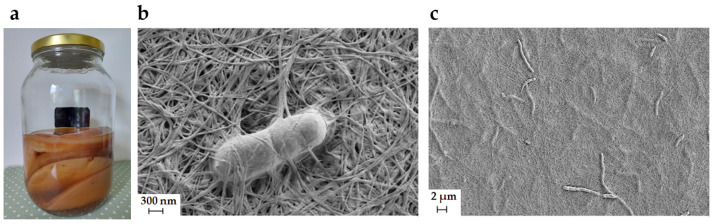
(**a**) Homemade kombucha tea with a visible mass of BC; (**b**,**c**) micrographs of *Komagataeibacter melomenusus* embedded in a cellulose network, taken with the scanning electron microscope.

Although cellulose is widely known, even outside the scientific community, to be the main structural component of many plant tissues, the biosynthesis of this homopolysaccharide not only takes place in plant cells, but similar anabolic reactions also occur in the world of microorganisms [1,2]. Prominent cellulose-producing microorganisms include certain algae, the Gram-positive bacterium *Sarcina ventriculi*, and a variety of Gram-negative genera such as *Achromobacter*, *Azotobacter*, *Pseudomonas*, *Rhizobium*, and *Salmonella* [3]. Some of the best-known producers of bacterial cellulose (BC) undoubtedly belong to the genera of acetic acid bacteria (AAB)—rod-shaped, Gram-negative obligate aerobes classified in the family *Acetobacteraceae* [4,5]. These α-*Proteobacteria*, capable of oxidizing alcohols, aldehydes, sugars, and sugar alcohols to acetic acid in the presence of oxygen, are of great economic importance [6]. In the food industry, they enable the production of vinegar, kombucha tea (Figure 1a), and nata de coco. Due to their high productivity and rapid biosynthesis, they are considered primary commercial BC-producing bacteria [7]. Two genera, *Komagataeibacter* and the recently described *Novacetimonas*, are characterized by their tolerance to low pH, efficient conversion of ethanol to acetic acid, and secretion of large amounts of BC [8,9]. Both genera occur in nature mainly as colonizers of fruits. The AAB species *Komagataeibacter europaeus*, *Komagataeibacter medellinensis*, *Komagataeibacter rhaeticus*, *Komagataeibacter xylinus*, and *Novacetimonas hansenii* are considered the best cellulose producers among bacteria and serve as model organisms for studying BC and its applications [10,11,12].

Microbial cells surrounded by cellulose fibers (Figure 1b,c) form a biofilm. This defensive layer can protect organisms from lack of water and the associated desiccation, damage caused by ultraviolet radiation, unfavorable pH conditions, and the accumulation of toxic substances [1]. BC is not essential for survival, but it gives the microorganisms that produce it a competitive advantage by supporting attachment, adherence, and following colonization of substrates [3].

**Figure 2:**
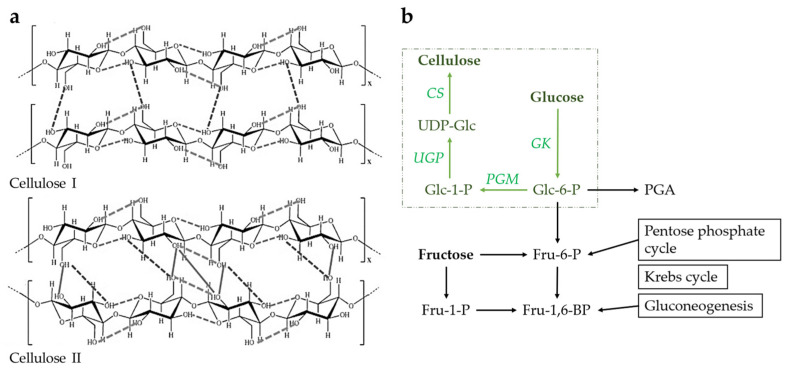
(**a**) Different allomorphs of cellulose: cellulose I (**top**), and cellulose II (**bottom**) (adapted from Ferro et al. [15] (CC BY)); (**b**) schematic representation of the biosynthetic BC pathway with steps from glucose to cellulose in green (GK—glucokinase, PGM—phosphoglucomutase, UGP—UDP-glucose pyrophosphorylase, CS—cellulose synthase).

Although the molecular formula of bacterial and plant cellulose is identical—both are biopolymers composed of D-glucose monomers linked by β-1,4-glycosidic bonds—these two types of cellulose differ in their microfibrillar structure [13]. The *Komagataeibacter* can biosynthesize two allomorphic forms of cellulose (Figure 2a) with the dominant cellulose I allomorph, which is less stable and more crystalline, and the cellulose II allomorph, which is thermodynamically more stable and present in a smaller proportion [14].

The biosynthetic BC pathway consists of four crucial enzymatic steps when glucose molecules are a carbon source (Figure 2b). These include glucose phosphorylation to glucose-6-phosphate (Glc-6-P) by glucokinase, Glc-6-P isomerization to glucose-1-phosphate (Glc-1-P) by phosphoglucomutase, and UDP-glucose (UDP-Glc) synthesis by UDP-glucose pyrophosphorylase [13]. The resulting UDP-Glc is a monomer that polymeryzes into a series of β-1,4-glucan chains. The cell immediately secretes these chains into the external environment, where they first self-assemble into fibrils and then into larger cellulose fibers. BC biosynthesis and export are primarily controlled by the *bcsABCD* operon, which encodes four major proteins comprising the bulk of cellulose synthase. The BcsA protein catalyzes the polymerization of UDP-Glc units and enables allosteric regulation of BC synthesis by cyclic di-GMP. Together with the BcsB protein, it forms a pore in the cytoplasmic membrane. The periplasmic protein BcsC forms a *β*-barrel pore in the outer membrane through which the growing glucan chain enters the external environment. Another periplasmic protein called BcsD controls the correct orientation of the cellulose synthase complex. The molecular mechanisms described allow polymerization at up to 200,000 glucose molecules per second [16,17].

**Figure 3:**
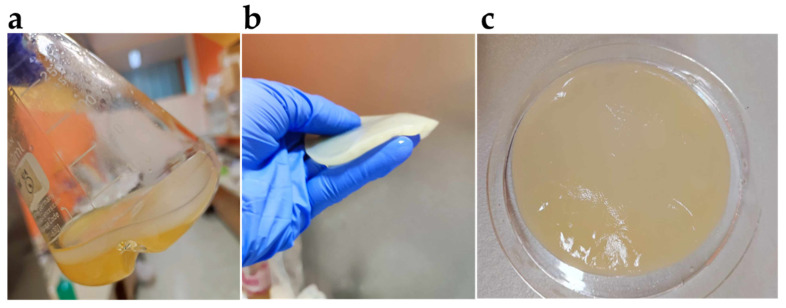
(**a**) Pellicle formed by *K. xylinus* in static culture; (**b**) bacterial pellicle—elastic and conformable, yet very robust material; (**c**) typical cellulose pellicle has a high water-holding capacity and possesses hydrogel-like properties.

Cellulose nanofibers, typically 25–100 nm in diameter and several micrometers in length, intertwine in large numbers to form a structure known as a cellulose lattice. As new fibers accumulate, the cellulose network increases in volume and density, eventually forming a mat visible to the human eye—a so-called pellicle [16]. BC-producing bacteria can be grown in static or agitated cultures. In the first case, bacteria grow at the liquid–air interface, where a higher oxygen concentration allows high-quality aerobic growth, forming a pellicle (Figure 3a). In the second case, where oxygenated air is added directly to the liquid growth medium, the cellulose fibers form millimeter-scale pellets, spheres, or a fibrous suspension with irregular shape [18].

Its unique physicochemical properties, which include a high degree of crystallinity, high tensile strength, excellent permeability, high water content, hydrophilicity, high porosity, large specific surface area, high elasticity, and conformability (Figure 3b), low density, high degree of polymerization, high purity, biocompatibility, and biodegradability, make BC an exciting platform for materials science [19].

The individual glucan chains are linked by van der Waals forces and intra- and intermolecular hydrogen bonds that form between the hydroxyl groups of the glucose monomers. This promotes the parallel stacking of cellulose molecules into crystalline nanofibers, which further assemble into cellulose nanofibrils. The strong molecular bonds contribute to a high degree of crystallinity and high tensile strength. A single BC nanofiber has a tensile strength of at least 2 GPa and Young’s modulus of about 138 GPa. BC also has a high water-holding capacity due to the specific arrangement and large number of hydroxyl groups in the glucose molecules, enabling the cellulose fibers to bind many water molecules. Hydrated BC has a very high water content (>98% *w*/*v*) and exhibits hydrogel-like properties (Figure 3c) [2,14].

The main advantage of BC over plant-derived cellulose is its high purity, as BC-producing bacteria do not co-produce other substances (e.g., hemicellulose, lignin, and pectin) alongside cellulose. The absence of these heterologous polymers and other impurities makes BC biocompatible. Plant-derived cellulose requires pre-processing, an energy-intensive and environmentally harmful purification process that involves numerous mechanical and chemical separation steps. BC produced by fermentation usually contains only traces of impurities (mainly microbial cells and their components, or the culture medium components), which significantly simplifies the purification process. In most cases, BC is purified using alkalis such as potassium hydroxide and sodium hydroxide or organic acids such as acetic acid, or it is repeatedly washed in a reverse osmosis process [13].

The market demand for BC is steadily increasing, and its commercialization is exponentially growing due to the wide range of BC applications in various sectors. In 2023, the global market for BC was estimated to be USD 480.1 million, and the expected market value is projected to reach USD 608.71 million by 2025 and USD 771.76 million by 2027, with a compound annual growth rate (CAGR) of 12.6% from 2019 through 2027 [20]. Despite BC production being capital-intensive, the research focus is on new, optimized production approaches aimed at cost reduction. These approaches include, but are not limited to, investigating new, low-cost media, discovering new bacterial strains, and utilizing synthetic biology tools to enhance production yields or achieve new properties.

### 2.2. Growth Media for Bacterial Cellulose Production

Human civilization has relied on various natural materials for thousands of years. In recent times, known as the age of advanced materials, society has been striving toward green technologies that significantly emphasize the circular economy—a new driver of innovation. Due to the emerging trend of more sustainable production processes, cost-effective yet efficient growth media are at the forefront.

BC is industrially exciting because it can be obtained quickly, inexpensively, and sustainably. Although the glucose in the growth medium for bacteria cultivation is the preferred carbon source for BC synthesis, other sugars, such as xylose, lactose, galactose, mannose, arabinose, fructose, and sucrose present in different agricultural and food waste materials, and industrial byproducts can also be used [21,22,23,24]. The efficiency of BC production on carbon sources other than glucose is different, but also, in the case of being lower, it is still attractive due to low or zero media prices and companies’ increasing awareness of the circular economy importance.

The most widely used synthetic complex growth medium for BC production is the Hestrin–Schramm (HS) medium, with glucose as a carbon source for bacterial growth and BC synthesis [25]. The BC production yield in this medium and media prepared from agricultural and industrial organic waste materials depends on the bacterial strain, the vessel used for bacteria cultivation, oxygen supply, pH of the medium, and cultivation time [2]. Depending on these parameters, the cellulose pellicle forms within a few (3–14) days and in high yields, reaching up to 10 g of dry weight per liter [22,23,26,27].

During the last 10 years, many research studies have been performed using different natural raw organic materials for BC production. The preferred natural organic material for BC production varies among countries depending on the climate suitable for growing fruits and grain types. Very appropriate raw organic waste materials are fruits since they contain high sugar content, but also since the medium can be easily prepared in a blender with the addition of some water followed by filtering and centrifugation. Jozala et al. [28] achieved an extremely high 60 g/L dry BC yield after 4 days of *Komagataeibacter xylinus* cultivation in a medium prepared from rotten fruits composed of plums, green grapes, pineapples, and apples. From industrial residues of the cashew apple juice processing, the BC was, after centrifugation, also directly produced at 3.1 g/L dry BC yield in 12 days of static cultivation [29]. When using industrial beverage waste material, such as fruit peels and pomace, more harsh conditions are necessary for preparing growth media, including enzymatic treatment or acid hydrolysis. Fan et al. [26] reported producing a 5.7 g/L dry BC yield after 9 days in medium from enzymatically treated citrus peel and pomace. Potato peel waste acid hydrolysate was also successfully used for BC production at 4.7 g/L dry yield after 6 days of cultivation [30]. Recently, Gorgieva et al. [23] prepared BC membranes using a novel species, *Komagataeibacter melomenusus*, in grape pomace hydrolysate in only 4 days with a 1.2 g/L dry BC yield. Also, the liquid industrial waste byproducts can be used directly, without any treatment, and as a sole medium without any supplements, such as cheese whey [31], thin stillage, which is a liquid byproduct that remains after microbial ethanol fermentation of carbohydrates by yeast and subsequent distillation of the fermented mash [22], and olive oil mill wastewater [32]. Other liquid waste materials, such as corn-steep liquor and laundry effluent for jeans processing, were also used directly but just as a replacement for a part of the synthetic HS medium [33]. The BC can also be produced in hot-water-extracted wood, a residual material originating from pulp mills and lignocellulosic biorefineries, although at only 0.15 g/L dry BC yield after 10 days of static cultivation [21], or glucose obtained from algae hydrolysate at 1.1 g/L dry BC yield after 7 days of static cultivation [27]. Tobacco waste extract was, after nicotine removal, also successfully used for BC production at 2.27 g/L dry BC yield after 7 days of static cultivation [24]. 

### 2.3. Application Niches of Bacterial Cellulose

**Figure 4:**
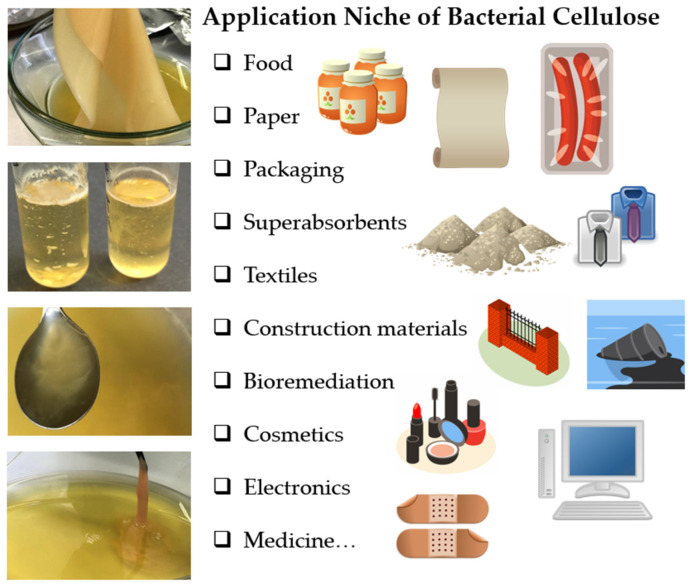
BC’s remarkably wide application niche, stemming from its unique and convenient physicochemical properties, encompasses diverse areas, including the food industry, superabsorbent-polymers market, production of sustainable microbial textiles, and biomedical engineering.

BC possesses many applicable properties, making this nanomaterial attractive in diverse areas, from the food and paper industry to the cosmetic industry and medical engineering (Figure 4). In general, the commercial niche of BC is guided by the shape and macro-appearance of the produced material, which depends on the fermentation method. The agitated fermentation ends up with hydrocolloid-like material used in the food industry as a thickener or suspending agent. In contrast, the pellicle-like material obtained at static cultivation is useful in the medical, pharmaceutical, and cosmetic fields and packaging [34].

#### 2.3.1. Application of Bacterial Cellulose in Food and Cosmetics

BC has immense potential in the food industry due to its ultra-pure, low-calorie nature and ability to hold large amounts of water. As a naturally occurring food rich in dietary fiber, it benefits the consumer’s health by reducing the risk of developing chronic diseases such as diabetes, obesity, and cardiovascular diseases [35]. In recognition of its safety, The United States Food and Drug Administration (FDA) granted BC the status of “generally recognized as safe” (GRAS) in the year 1992, enabling numerous FDA-approved products to use its convenient material properties [36].

**Figure 5:**
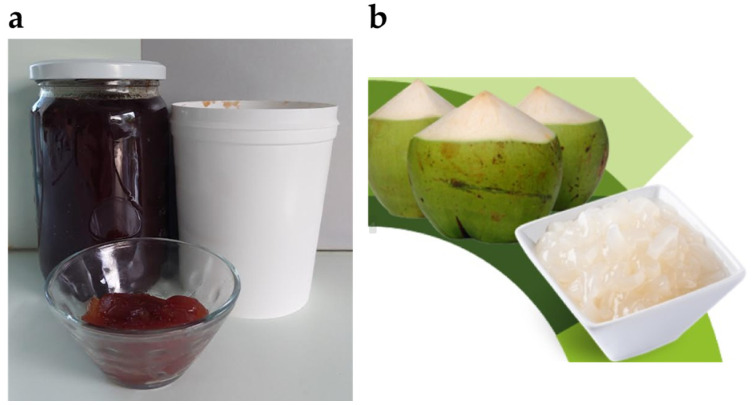
(**a**) Because of its low-calorie properties, BC is often a fat substitute in meat or bulking agent in jam; (**b**) coconut fruits and gelatinous cubs of nata de coco—a sweet Asian dessert based on BC (adapted from Rachtanapun et al. [39] (CC BY)).

Unprocessed BC is tasteless and has a rigid structure, but it can be softened by treatment with alginate, calcium chloride, or sugar alcohols. Due to having excellent water-holding properties and not compromising structural integrity, BC is often added to processed foods to help maintain their original properties for extended periods. With its gelling, thickening, stabilizing, suspending, and emulsifying ability, cellulose is a suitable candidate for various food products such as pastries, salads, and yogurts. A significant area of the food industry is diet-food production, where BC is frequently included as a quality, low-calorie substitute for fats (Figure 5a) [35]. Recent toxicological experiments in mice related to acute, subacute, and subchronic oral toxicity assays after consumption of BC demonstrated the absence of reproductive toxicity, embryotoxicity, teratogenicity, and genotoxicity effects [37]. Another comprehensive review on the toxicology and dietetic role of BC when tested on animals and in vitro [38] also showed that BC is not toxic after ingestion, it is not carcinogenic, tumor-promoting, pyrogenic, or a developmental or reproductive toxicant and thus it is not expected to pose any adverse side effects when used in human foods.

The beginning of BC utilization in food industry dates back to the 1960–1970s in the Philippines, for producing healthy coconut gel, known as nata de coco (Figure 5b), where the BC is formed in the sugar syrup. This sweet dessert is commercially produced in several Asian countries, including Malaysia, Thailand, and Vietnam, as well as the Philippines. The production process of this cube-shaped delicacy involves the acetic acid bacterium *K. xylinus* metabolizing various carbohydrates, coconut water, and amino acids. Nata de coco is exploited in drinks, yogurts, pies, salads, and sausages, bringing a new flavour or texture to the formulations [40,41].

The use of various probiotics capable of enhancing gut microbiota and improving digestion has recently been rising. However, their usefulness is significantly limited by a short shelf life and instability. One possible solution to this problem is the encapsulation of probiotics with biocompatible and antibacterial materials, capable of enhancing the stability of probiotics over a long period, improving their resistance to adverse manufacturing conditions, and protecting them from the acidic conditions of the human digestive system. Due to its unique physicochemical properties, especially high crystallinity, biocompatibility, and non-toxicity, BC is highly suitable for encapsulating probiotics [35]. For instance, Fijałkowski et al. [42] showed that BC is a good carrier for immobilizing probiotic *Lactobacillus* spp., as it provides high-level protection of the microorganisms against the influence of gastric acid juice and bile salts, while Oliveira-Alcântara et al. [43] prepared BC/cashew gum films with probiotic *Bacillus coagulans* and/or prebiotic fructooligosaccharides and observed very good storage stability of probiotics. Aside from application in the gut, a recent study demonstrated the effectiveness of BC containing *Lactobacillus fermentum* and *Lactobacillus gasseri* against pathogens on skin [44]. Here, it strongly reduces the viability of *Staphylococcus aureus* and *Pseudomonas aeruginosa*, the most active pathogens in severe skin infections and chronic wounds and inhibits the proliferation of methicillin-resistant *S. aureus*.

BC also has significant potential in the cosmetic industry due to its valuable properties such as biocompatibility, water-holding capacity, adhesiveness, absorption, and release of substances. As a sustainable material, it could replace many non-biodegradable cosmetic ingredients. Among the commercially available cosmetic BC products, skin masks are particularly well-represented. Membranes made from BC nanofibers can incorporate a wide range of cosmetic actives (for example, moisturizers and astringents), allowing these skin masks to not only hydrate and moisturize the skin but also serve as carriers for various active ingredients [45]. In addition to skin masks, cellulose makeup pads and BC-derived cosmetic additives are also available [2].

#### 2.3.2. Application of Bacterial Cellulose as Advanced Material

Deforestation is a pressing issue in the conventional paper industry and has led to the intensive search for alternative methods of paper production using sustainable sources. Recyclable paper and paper pulp based on herbaceous (non-woody) plant fibers are becoming increasingly popular. The current challenge lies in the superior quality of traditionally produced paper compared to recycled paper, as the latter often fails to maintain the original physical and mechanical properties. One of the alternatives includes utilization of BC, which can enhance the mechanical properties of the materials it is merged with [46]. Therefore, it represents a suitable candidate for the sustainable production of high-quality paper pulp and paper [47]. Basta and El-Saied [48] demonstrated that adding modified BC, produced in the culture medium in the presence of glucose phosphate, to the wood pulp significantly improves kaolin retention, strength, and fire-resistance properties of the resulting functional paper. A recent mini review [49] provides a summary of specialized types of papers, containg BC as a modifier, such as conductive, fluorescent, and fire-resistant papers.

The inevitable consequence of the exponential growth of the human population is the persistent increase in consumption, followed by greater demand for products and services, as evidenced by the widespread use of plastic packaging. Petrochemically produced synthetic plastic has numerous adverse effects, particularly concerning its accumulation and incineration, leading to a growing focus on reducing and potentially eliminating such packaging in favor of biodegradable packaging materials. Plant-derived cellulose currently dominates in green packaging, which often relies at least partially on cellulose fibers. However, BC fibers offer a more reasonable alternative, as they are inherently ultra-pure and do not require pre-processing, resulting in lower production costs [50,51].

In their dry state, superabsorbent polymers can spontaneously absorb a volume of liquid twenty times their weight (a 2000% change in volume) while retaining their original identity. Currently, the majority of superabsorbents are produced from non-renewable and non-biodegradable polymers. This creates a significant market potential for biodegradable and natural polymers with high water-holding capacity, such as BC. Commercial superabsorbent polymers are found in baby diapers, adult incontinence products, and female hygiene products and are used in agriculture for the controlled release of fertilizers [35,52].

**Figure 6:**
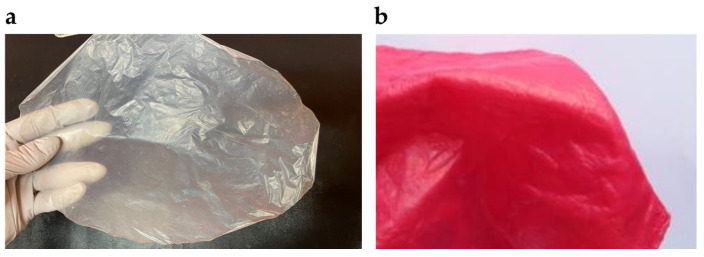
(**a**) As dried BC membranes are inherently beige, the dyeing process is an essential yet often ecologically damaging step in BC textile production; (**b**) a BC composite dyed with Procion Red H-E3N reactive dye (adapted from Fernandes et al. [54] (CC BY)).

Due to new environmental policies, sustainable post-petroleum synthetic textiles originating from bacteria and fungi are gaining importance. The textile and leather industries are among major environmental polluters—agricultural production and industrial processing contribute to greenhouse gas emissions, and tanning and dyeing contaminate water. At the same time, synthetic fibers gradually break down into microplastic particles. Natural biomaterials, such as hair and skin, use their cells to produce pigments, thus sustainably coloring the material. In contrast, the analogous process of industrial textile dyeing (Figure 6a,b) requires a series of chemical reactions and has a significant environmental impact. Alternative dyeing methods must be explored to achieve fully sustainable green textiles, including BC-derived ones. For instance, self-dyeing BC produced by a strain of genetically engineered acetic acid bacteria that synthesize melanin due to recombinant tyrosinase expression holds excellent potential [53].

There is a growing demand for sustainable construction materials with multifunctional properties. Nanoparticles have been popular for several decades for enhancing cement hydration, serving as reinforcement, and densifying microstructures, leading to reduced porosity and increased mechanical strength [55,56]. Green, non-toxic, and multifunctional nanocellulose materials hold a special place within the class of nanoparticles. Among them, plant-based cellulose nanofibers are widely known. Additionally, bacteria-derived cellulose, produced through uncomplicated fermentation and with a more cost-effective production process, represents a favorable alternative for obtaining bioconcrete [57].

Bioremediation, specifically microbial remediation, involves using various microorganisms to transform pollutants into simpler molecules such as carbon dioxide and water. It is a practical approach for containing and detoxifying different forms of contamination. There are several bioremediation applications of BC: modified BC adsorbs specific contaminants [58], cellulose-based ultrafiltration membranes filter polluted liquids [59], and composites of BC and other materials degrade pollutants [60].

In consumer electronics, many prominent developers and manufacturers follow a marketing strategy focusing on developing sustainable and flexible devices. BC is being used to achieve sustainability and recyclability, as it is a quality natural biopolymer with outstanding biodegradability, mechanical performance, piezoelectricity, and dielectricity [35,61]. One of the notable processing techniques is the carbonization of BC, where the entire cellulose structure is transformed into a highly conductive carbon network, resulting in carbonized BC (CBC) [62]. Fully or at least partially BC-based devices and their components include electronic paper, transistors, flexible electrodes, organic light-emitting diodes (OLEDs), digital displays, battery separators, and acoustic diaphragms for headphones and speakers [63]. BC-made commercial products also include biodegradable deformation sensors, essential sensor devices in virtual and augmented reality recreational markets, and gaming. Several major electronics companies, including Acer, Audioquest, Creative, Klipsch, Panasonic, and Sony, sell headphones equipped with BC-based diaphragms in the consumer electronics market [35].

An emerging field concerning BC production, engineered living materials (ELMs), has recently evolved. Natural biomaterials possess various unique and valuable properties that interest materials science, and organisms that produce such materials can transform simple raw materials to form large and complex natural biomaterials. However, their properties are restricted to the evolved set of genetic information. The emerging field of ELMs addresses these limitations, dedicated to constructing BC-based engineered living materials as well [64]. ELMs, which lie at the intersection of microbiology, materials science, and synthetic biology, utilize molecular biology tools to reprogram living cells at the DNA molecule level [53]. Broadly, ELMs can be defined as dynamic and responsive materials with programmable properties, where genetically engineered cells comprise an integral part [36]. The living biological entities that shape ELMs are responsible for their fabrication, assembly, and functionalization [14]. The foremost goal is to create new or enhanced biomaterials tailored for specific applications in textile, fashion, and later also in other industries.

#### 2.3.3. Application of Bacterial Cellulose in Medicine

**Figure 7:**
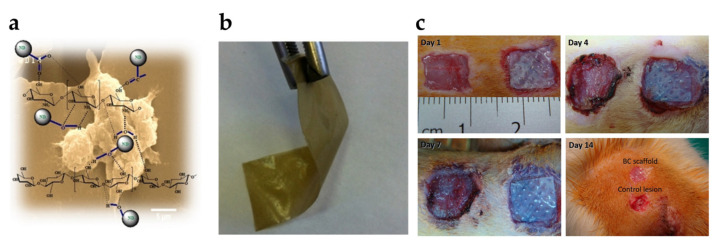
(**a**,**b**) Chitosan/BC composite films with diamond nanoparticles are among numerous BC-derived platforms for wound dressings (reproduced from Ostadhossein et al. [67] (CC BY)); (**c**) comparison of wound recovery on a rat model without (**left side**) or with (**right side**) BC bio-scaffold covering (reproduced from Cherng et al. [68] (CC BY)).

Due to their useful physicochemical properties such as exceptional safety, high permeability, high water-holding capacity, excellent flexibility, pronounced biocompatibility, and biodegradability, BC-made dressings and bandages have great potential in wound and burn treatment [65] (Figure 7a,b). High-quality materials used for wound recovery are known to be non-toxic, non-allergenic, maintain a moist wound environment, absorb exudates, enhance re-epithelization, exhibit high porosity and gas permeability, and are suitable for functionalization with antibiotics, as well as convenient for drug delivery [13]. Since wound recovery involves complex interactions among various cell types, soluble compounds and extracellular matrix components, the candidate material should be an appropriate interface to facilitate this process [66] (Figure 7c).

The still predominant clinical approach, in which wounds are dressed with antibiotic-containing dressings, can be expensive, ineffective, and may contribute to the development of bacterial resistance to antibiotics [65]. Alternatively, BC, which does not possess intrinsic antimicrobial properties in its natural state, can be functionalized with antimicrobial substances [69]. Some examples are therapeutic metals and antibacterial nanomaterials (e.g., copper, silver, zinc, zinc oxide, titanium dioxide, and montmorillonite), natural polymers (e.g., chitosan), synthetic polymers (e.g., polyethyleneimine), and different antibiotics (e.g., amoxicillin).

In combination with other materials, BC is suitable for controlled drug delivery. Graphene nanoparticles are appropriate drug carriers capable of preventing the premature release of active substances. Since they tend to aggregate in aqueous solutions, embedding them in a BC network may offer a proper solution [70]. As a material enabling prolonged drug release, BC is a convenient carrier for therapeutic agents in cancer treatment. BC allows for controlled and localized chemotherapy, resulting in a higher drug concentration at the tumor site while minimizing exposure to surrounding tissues and reducing unwanted side effects [71,72]. Modified BC can trap cancer cells by implanting cellulose membranes functionalized with chemoattractants at the site of tumor removal [73].

Using various BC composites as scaffolds in tissue engineering is becoming increasingly common. Some pioneering ideas include 3D porous microspheres made from BC and collagen [74], BC with plant-derived cellulose nanocrystals and hydroxyapatite added in situ [75], and BC incorporated with the bone phosphoprotein osteopontin [76]. Gorgieva and Hribernik [77] proposed a combination of BC with microstructured gelatin to develop scaffolding membrane for guided tissue regeneration. The biocompatibility, fine architecture of cellulose fibers, and excellent tensile strength of BC enable the production of artificial blood vessels. Furthermore, due to its ability to promote neovascularization, BC is suitable for stimulating the growth of new native blood vessels [78].

## 3. Strategies for Improvement of Bacterial Cellulose Production and Modification

### 3.1. Conventional Methods for Improvements of Bacterial Cellulose Production and Modification

**Figure 8:**
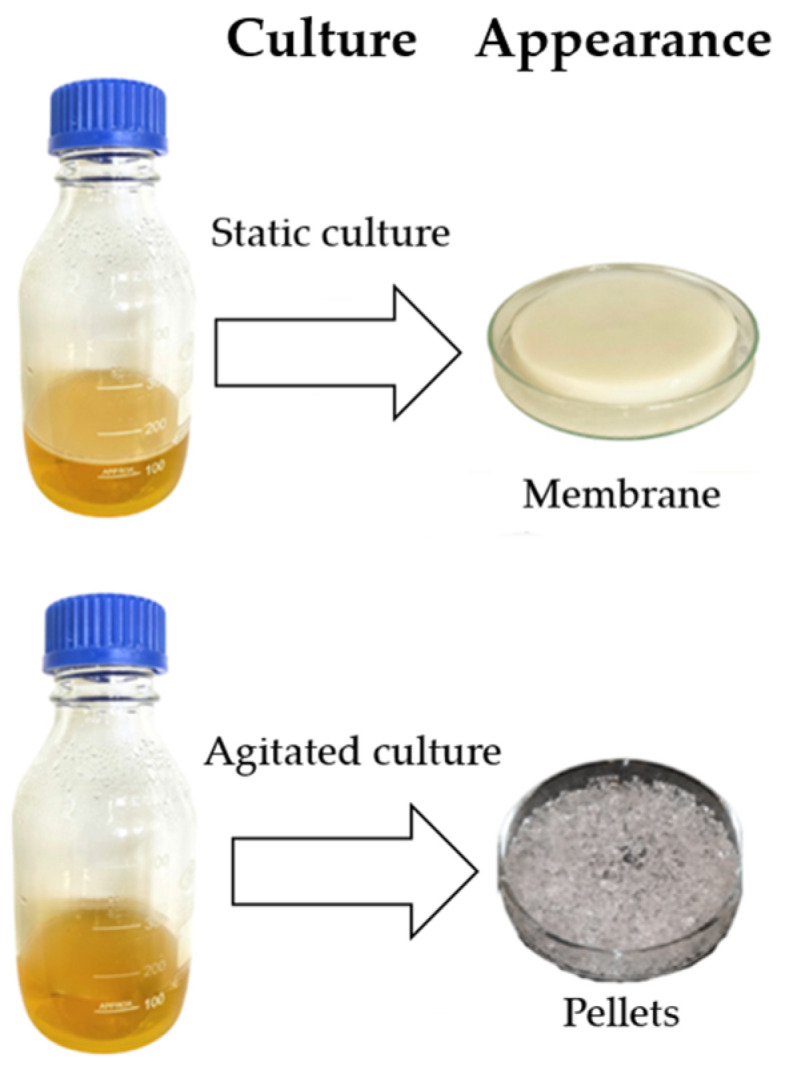
BC’s final appearance depends on the fermentation technique, with static culture (**top**) resulting in BC membranes (pellicles) that form at the liquid–air interface and assume the shape of the laboratory vessel, and agitated culture (**bottom**) leading to the submerged formation of irregular BC pellets (adapted from de Amorim et al. [85] (CC BY)).

Applications and commercialization of BC were initially significantly limited by low yields in laboratory fermenters and inefficient scale-up of the production process to an industrial scale, as economically justified yields were not achievable in industrial bioreactors [14]. Consequently, researchers dedicated several years of studies to optimizing bioprocess factors by modifying culturing strategies and conditions. They investigated the influence of essential medium components, carbon sources, various additives, temperature, pH, dissolved oxygen concentration, dimensions of laboratory vessels, and different fermentation techniques, focusing on static and agitated cultures (Figure 8) and bioreactors [79,80,81,82,83,84].

Static culture—the most popular strategy for producing BC, in which microorganisms form a cellulose pellicle at the liquid–air interface—is limited by long cultivation times. This drawback can be overcome by the agitation fermentation technique, where bacteria are incubated on a shaker, resulting in BC with a similar chemical structure. Although bacterial growth in agitated culture is higher than in static culture, shaking can lead to the occurrence of mutants in cellulose operons, which do not contribute to the final BC yield [14].

Researchers have attempted to improve the original medium to optimize cellulose production by adding a cheaper nitrogen source, such as corn steep liquor, incorporating various sulfates and phosphates, and removing organic acids [86,87]. The carbon source is one the most extensively studied components of the growth medium, as it significantly affects the BC yield. BC-producing bacteria vary in their ability to assimilate and utilize different carbon sources [14]. To enhance the BC yield, a wide range of primary metabolites have been tested, including various types of carbohydrates such as monosaccharides (e.g., D-xylose, D-xylulose, fructose, galactose, glucose), disaccharides (e.g., lactose, maltose, sucrose), oligosaccharides, organic acids, and sugar alcohols (e.g., ethanol, mannitol) [88,89,90,91,92,93,94,95].

Before the introduction of genetic engineering techniques in BC production, the artificial selection of strains was considered a relatively simple genetic intervention capable of influencing the production process. Various species of bacteria used in industrial BC production exhibit different yields depending on the growth medium and culturing conditions. They also differ in the properties of the final product, particularly in its purity and crystallinity. Artificial selection, combined with naturally occurring mutations, allowed researchers to identify the most successful strains with the highest product yield and quality for each species [7,96,97]. While such a non-targeted approach can be successful, it cannot gain the speed and precision of genetic engineering. Furthermore, it is unsuitable for introducing new functionalities into microorganisms since the necessary genes are commonly missing [12].

Like any natural or synthetic material, BC has limited usability, which can be expanded through various conventional functionalization methods. Based on the approach of modifying the material, three types of cellulose are distinguished: bacterial nanocellulose (BNC), cellulose nanocrystals (CNCs), and cellulose nanofibrils (CNFs). BNC is produced through a bottom-up approach by bacteria metabolizing low molecular weight carbohydrates [98]. Applying the top-down approach to BNC enables the production of CNCs and CNFs. CNCs are formed through treatment with chemicals, such as ionic liquids, lithium hydroxide, N,N dimethylacetamide/lithium chloride, N-methylmorpholine-N-oxides, and NaOH-urea/thiourea [99,100,101,102,103]. CNFs are obtained through mechanical processes, such as high-pressure homogenization and grinding combined with enzymatic pretreatments [104,105]. Particular challenges accompany the production of cellulose nanomaterials. The limited selection of suitable solvents leads to the use of toxic and hazardous chemicals, which pollute the environment and limit the recyclability and disposal of products. Meanwhile, mechanical processing methods may compromise the physical properties of the biopolymer.

Conventionally, without using genetic engineering methods, numerous composites can be produced where BC is combined with other materials. In principle, we can distinguish two different strategies. In the first strategy, the in situ strategy, various reinforcement materials are added directly into the growth medium and incorporated by microorganisms during the biosynthesis of BC. Commonly added substances capable of improving the functional properties of BC include agar [82], aloe vera [106], carboxymethyl cellulose [107], conductive polymers (polyaniline, PEDOT:PSS, polypyrrole) [108], graphene oxide [109], metal nanoparticles [110], multiwalled carbon nanotubes [111], poly-3-butyrate [112], and sodium alginate [113]. Some issues associated with this kind of strategy include the precipitation of added substances and the unsuccessful incorporation of reinforcement materials into the pellicle. In the second strategy, the ex situ strategy, physical absorption or hydrogen bond formation is employed to establish connections between additives and the cellulose matrix. Such nanocomposites are often developed by combining BC with carbon nanotubes, chitosan [114], collagen [115], conductive polymers [116], hyaluronic acid [117], hydroxyapatite [118], metal nanoparticles [119], metal oxides [120], and montmorillonite [121]. The use of these reinforcement materials enhances the mechanical, electrical, and antimicrobial properties of BC. The utility of this strategy is mainly restricted by the non-uniform size of additives and cellulose pores, as well as the hydrophilic nature of BC, which hinders interactions with hydrophobic reinforcement materials [14].

**Figure 9:**
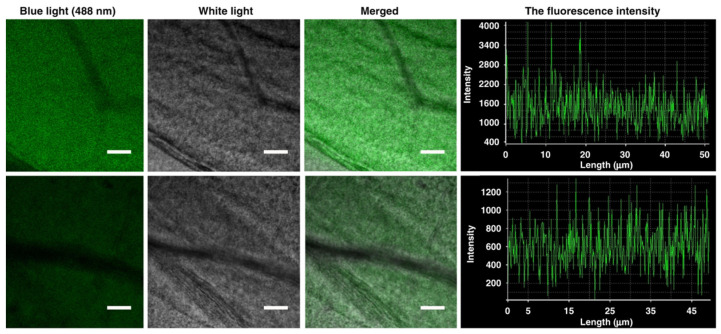
The fluorescence intensity of functional BC grown in the presence of 6-carboxyfluorescein glucose (6-CF-Glc) depends on the substrate concentration; a high concentration of 6-CF-Glc (**top**) and low concentration of 6-CF-Glc (**bottom**) with average fluorescence intensities of 1492 a.u. and 612 a.u., respectively (adapted from Gao et al. [122] (CC BY)).

Gao et al. [122] showed the production of functional BC by *K. sucrofermentans* where instead of genetically engineering the microorganisms, they modified glucose into synthetic 6-carboxyfluorescein glucose (6-CF-Glc). The bacteria grown in the modified glucose medium synthesized a cellulose pellicle with unnatural green fluorescing capabilities (Figure 9). The fluorescence intensity was controlled by varying the concentration of 6-CF-Glc in the growth medium.

### 3.2. Co-Culturing Bacteria for Improvements of Bacterial Cellulose Production and Functionality

**Figure 10:**
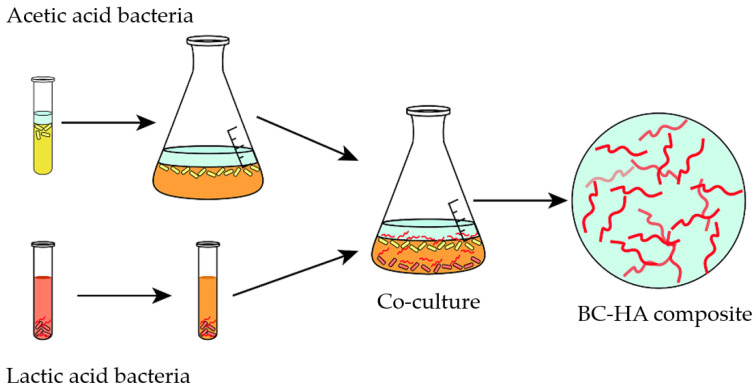
Schematic representation of co-culture approach for obtaining BC and hyaluronic acid (HA) composite (adapted from Brugnoli et al. [128] (CC BY)).

Several examples exist of using the co-culture approach for BC production (Figure 10). In one of the early studies, *K. xylinus* and *Lactobacillus mali* were co-cultured in corn- steep liquor supplemented with sucrose [123]. The authors noted a 3-fold improvement in BC production compared to the monoculture of *K. xylinus*, due to cell–cell interactions and the exopolysaccharide produced by *L. mali*. BC made in *N. hansenii* and *E. coli* ATCC 700728 co-culture displayed superior mechanical properties [124]. In the co-culture of *N. hansenii* ATCC 23769 and *Lactococcus lactis* APJ3, a copolymer of BC and hyaluronic acid was successfully produced, showing improved water-holding capacity compared to regular cellulose pellicles [125]. Similarly, in the co-culture of *K. xylinus* and *Ralstonia eutropha*, a mechanically superior nanocomposite of BC and polyhydroxybutyrate was developed [126]. Gunduz et al. [127] expanded this approach by co-culturing *N. hansenii* ATCC 23769 with a consortium of microorganisms (e.g., *Lactobacillus casei*, *L. lactis*, *Rhodopseudomonas palustris*, and *S. cerevisiae*) that assists the display of multiple functionalities. They achieved a 40-fold increase in water-holding abilities compared to the monoculture of *K. xylinus*.

Taking a slightly different approach inspired by the symbiotic culture of bacteria and yeast (SCOBY) used for fermenting kombucha tea, Gilbert et al. [63] produced BC in a co-culture of genetically modified yeast and a wild-type strain of *K. rhaeticus*. They focused on genetically modifying the yeast *S. cerevisiae*, a well-known model organism that can be genetically manipulated in ways not possible with the *Komagataeibacter* genus. The authors initially aimed to genetically engineer the yeast, known for its excellent protein secretion capabilities, to secrete proteins that would functionalize the developing cellulose pellicle. They tested this concept using β-lactam hydrolyzing enzyme TEM1 β-lactamase, and apparent enzymatic activity was detected in the pellicles produced in the co-culture containing both BC-producing bacteria and genetically modified yeast. This activity was preserved even after the functionalized BC was dried and rehydrated. Furthermore, they designed a yeast strain that modifies pellicles’ physical properties. The microorganisms simultaneously secreted different cellulose-degrading enzymes, including β-glucosidase, cellobiohydrolases, endoglucanase, and lytic polysaccharide monooxygenases. They found that the BC production was not significantly reduced, but some of the pellicle’s physical properties, especially its tensile strength and Young’s modulus, were weakened. Co-culturing acetic acid bacteria and yeast was also used to investigate whether the living BC-based hybrid materials can sense and react. They designed a yeast biosensor system based on the synthetic transcription factor Z3EV and the reporter gene encoding the green fluorescent protein (GFP), whose transcription could be induced by the estrogen steroid hormone β-estradiol (BED). Since the cellulose pellicle grown in such co-culture displayed intense green fluorescence when exposed to externally applied BED, the authors concluded that the living BC-based hybrid materials can sense environmental stimuli and react correspondingly.

### 3.3. Genetic Engineering of Bacterial Cellulose Producing Strains

Although genetic engineering has been one of the critical tools in biotechnological research and industrial biotechnology since the 1980s, until recently, it was not extensively used for improving the production or modification of BC. This may be attributed to numerous non-genetic interventions that researchers and manufacturers employed effectively to enhance production, increase yield, modify the properties of the biopolymer, and introduce new functionalities to the resulting material. However, in the last two decades (Table 1), several examples proved that using genetic engineering of *Komagataeibacter* species can render additional improvements to BC production.

#### 3.3.1. Conventional Gene Targeted Approaches for Modification of Bacterial Cellulose Production

Initially, genetic modifications of *Komagataeibacter* spp. were confined to plasmid vector backbones, such as pSA and pBBR122, and focused on adding or removing genes to enhance BC production [13]. The first more significant demonstration of genetic engineering in cellulose-producing bacteria was reported in a study from 1999, where a DNA sequence encoding the sucrose synthase of mung bean (*Vigna radiata*) was introduced into *K. sucrofermentans* [129]. The genetically modified bacteria produced more BC than the wild-type strain when sucrose was present in the growth medium. Among the positive effects of the described genetic manipulation, which enabled acetic acid bacteria to metabolize sucrose, was increased BC yield and the possibility of using cheaper sucrose-based growth media.

An alternative method to improve BC yield using an inexpensive growth medium was developed by Battad-Bernardo et al. [130]. Their genetically modified bacteria could metabolize a low-cost lactose-based medium derived from whey. By inserting the *E. coli* β-galactosidase-encoding gene (*lacZ*) into *K. xylinus*, researchers achieved a 28-fold increase in BC production.

On the other hand, Chien et al. [132] aimed to enhance BC production by improving cells’ oxygen utilization. They induced the expression of the *vhb* gene, which encodes *Vitreoscilla* hemoglobin, in *K. xylinus* BCRC 12334. As a result, the genetically engineered strain showed better oxygen regulation than the wild-type strain. The expression of the *vhb* gene led to 50% higher cell growth and 20% higher BC production when the bacteria were incubated on a shaker.

During BC production, the cell oxidizes glucose to gluconic acid, leading to acidification of the growth medium, which in turn slows down BC production. Shigematsu et al. [131] attempted to address this issue by knocking out the *gdh* gene, which encodes glucose dehydrogenase, the enzyme responsible for converting glucose to gluconic acid. The authors reported a 1.7-fold improvement in BC production in the genetically modified strain compared to its wild-type counterpart.

As part of an extensive study to improve BC production, Jang et al. [138] used data from the sequenced genome and metabolome analysis of *K. xylinus* to construct a genome-scale metabolic model. Initially, they overexpressed two carbon metabolism-connected genes, *pgi* (coding for glucose-6-phosphate isomerase), and *gnd* (coding for phosphogluconate dehydrogenase), originating from *E. coli* or *Corynebacterium glutamicum*, resulting in a 2-fold increase in BC yield. In their model, they discovered that the intracellular level of adenosine triphosphate (ATP) plays a critical role in determining the BC yield. The enzyme glucose-6-phosphate dehydrogenase, encoded by the *zwf* gene, acts as a branching point where the cellular mechanism decides whether to metabolize glucose or use it for biosynthetic reactions. Since the activity of this enzyme is significantly inhibited by high levels of intracellular ATP, an increase in intracellular ATP concentration redirects more glucose molecules toward cellulose synthesis reactions. To maximize the intracellular ATP level, Gwon et al. [139] used a plasmid containing the *pfkA* gene, which encodes phosphofructokinase, an enzyme critical for glycolysis absent in the genus *Komagataeibacter*. Heterologous expression of the *E. coli pfkA* gene established the glycolytic pathway in *K. xylinus* (where glucose is usually metabolized via the alternative pentose phosphate pathway), resulting in a four-fold increase in intracellular ATP concentration, higher growth, and improved BC production.

**Figure 11:**
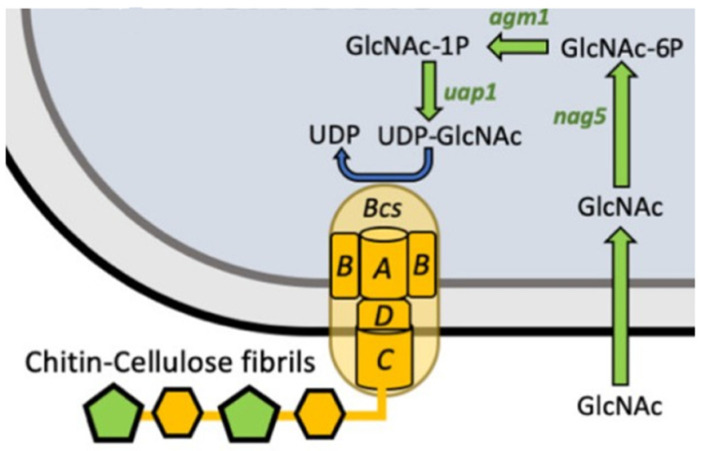
Schematic representation of cellulose/chitin copolymer production in genetically modified strain of *K. xylinus* expressing operon of three genes from *C. albicans* (GlcNAc—N-acetylglucosamine, UDP-GlcNAc–UDP-N-acetylglucosamine) (adapted from Singh et al. [12] (CC BY)).

In another significant study, Yadav et al. [133] introduced a *bla* promoter-controlled operon consisting of three genes from *Candida albicans* into *K. xylinus*, thereby altering the flow of cellular metabolites during the BC biosynthesis. This genetic intervention enabled the incorporation of the chitin monomer, activated cytoplasmic UDP-N-acetylglucosamine (UDP-GlcNAc), into the glucan chains alongside UDP-Glc. When both glucose and N-acetylglucosamine were available in the growth medium, the cellulose synthase of the genetically modified strain used UDP-Glc and UDP-GlcNAc to synthesize a copolymer of cellulose and chitin (Figure 11). The resulting cellulose/chitin copolymer exhibited improved in vivo degradability due to the susceptibility of chitin to degradation by enzymes in animal lysosomes. The authors also found that partial or complete glucose replacement with GlcNAc significantly reduced the final cellulose production.

The properties of produced BC were also investigated by Jacek et al. [142], who studied how the motility genes *motA* and *motB* influence the morphology of the cellulose pellicle in *N. hansenii*. Overexpression of the two genes resulted in a loosening of the intramembrane structure and fiber thickening. In contrast, disrupting the same genes, causing reduced mobility, led to a denser and more compact BC with improved mechanical properties.

Liu et al. [143] focused on improving another fundamental physicochemical property of BC, which is its water-holding capacity. Working on *Enterobacter* sp. FY-07, they induced the expression of the *wca* operon, carrying the genetic code for colanic acid, a water-soluble polysaccharide. By varying the concentration of the inducer, cellulose hydrogels differing in crystallinity, rheological properties, and water-holding capacity were developed. Their water-holding capacity was 1.7 times higher than that of the pellicle produced by the wild-type strain.

#### 3.3.2. Standardized Genetic Tools for Bacterial Cellulose Production with Advanced Characteristics

**Figure 12:**
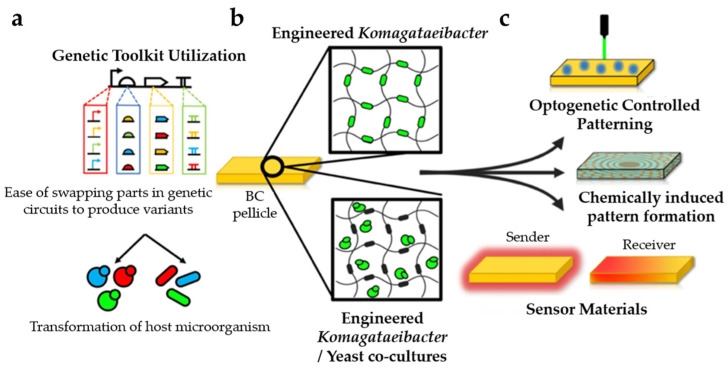
Overview of standard synthetic biology methods that are utilized for the development of living and functional BC-based hybrid materials; (**a**) synthetic libraries comprise modular DNA parts (including promoters, ribosome-binding sites (RBSs), coding sequences (CDSs), and terminators) which can be joined together into gene constructs for transformation into a host microorganism, such as yeast (circles) or *Komagataeibacter* (rods); (**b**) genetically modified cells secrete cellulose nanofibers into their surroundings and gradually form a BC pellicle within which they are contained; (**c**) BC pellicle encompasses living genetically modified cells that can sense and react as a response to selected externally applied signals, such as light, chemicals, and diffusible signaling molecules (adapted from Singh et al. [12] (CC BY)).

Florea et al. [136] were the first to develop a standardized genetic toolkit (Figure 12) for BC-producing bacteria, specifically for the *K. rhaeticus* iGEM strain isolated from a kombucha tea pellicle. Their study selected five replicative plasmid backbones, including pSEVA321, pSEVA331, pSEVA351, pBAV1K-T5-sfGFP, and pBla-Vhb-122. Their genetic toolkit also included reporter genes encoding various fluorescent proteins, terminator sequences, synthetic promoters of different strengths, and two types of regulated promoters. The inducible promoters were regulated by transcription factors, chemically inducible with anhydrotetracycline, which is an antibiotic analog, or N-acyl-homoserine lactone (AHL), a quorum sensing molecule involved in intercellular communication. They designed their genetic toolkit to a standardized cloning format named BioBricks, which allows for quick assembly of variously combined modular DNA parts in *E. coli* plasmids and subsequent introduction into *K. rhaeticus*. To demonstrate the usefulness of the new standardized genetic toolkit, the authors genetically engineered bacteria to respond to different concentrations of AHL. Afterward, they induced the production of red fluorescence only on one side of the developing pellicle or only in its newest layers, thus illustrating that spatial patterning is achievable with their tools. Furthermore, they replaced the module for red fluorescence with another one that expressed a synthetic RNA-based silencing system designed to repress the chromosomal gene for the enzyme UDP-glucose pyrophosphorylase, which is crucial for BC synthesis. As a result of this genetic intervention, they obtained a genetically modified bacterium that stopped producing a BC pellicle when a sufficient concentration of AHL was added to the growth medium.

**Figure 13:**
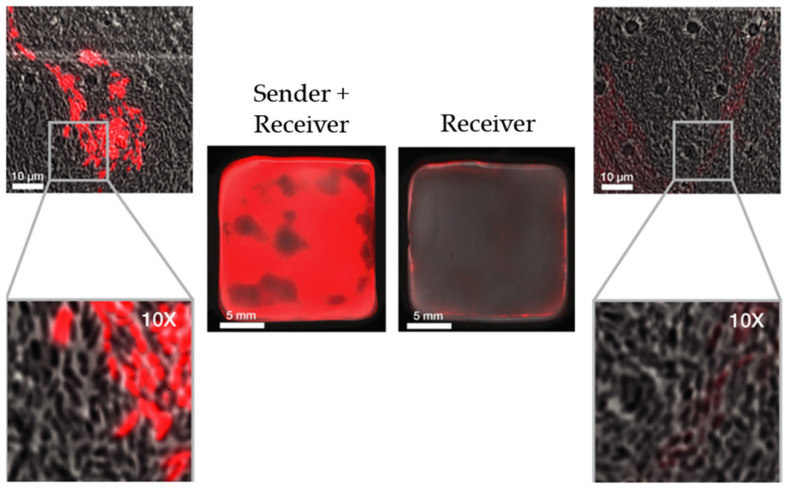
Comparison of red fluorescence response in the co-culture of sender and receiver cells (**left**) and monoculture of receiver cells (**right**) (adapted from Walker et al. [140] (CC BY)).

In the following study [140], the same group of researchers successfully demonstrated, through an innovative experiment, that the induction of gene expression in plasmids of the aforementioned bacterial cells can be controlled by other genetically modified cells grown in the same co-culture. The genetically engineered strain producing a red fluorescent protein (RFP) when induced with AHL, was combined with another recombinant strain capable of constitutively expressing the gene for the AHL-synthesizing LuxI enzyme. The new strain, consisting of the so-called sender cells, secreted AHL molecules into the medium where co-culture was grown. When these molecules reached a sufficient concentration and were near the so-called receiver cells, controlled by AHL, they initiated the production of RFP (Figure 13). Through this experiment, the researchers illustrated that the two genetically modified cells coexisted in co-culture while producing BC capable of creating red fluorescence. Perhaps more significantly, this study revealed that it is possible to grow materials that autonomously detect borders between different populations of genetically modified cells and induce gene expression only at those specific locations.

Teh et al. [141] extended the existing genetic toolkit by introducing several additional modular parts, such as new constitutive promoters and terminator sequences. They incorporated an arabinose-inducible promoter (P_BAD_) that reacts to high sugar concentrations (4%). Through different experiments, they demonstrated that the standardized genetic toolkit was efficient not only for *K. rhaeticus* but also for various strains of *K. xylinus* and *N. hansenii*. They also described programmable regulation of gene expression using the Clustered Regularly Interspaced Short Palindromic Repeats (CRISPR) approach. By employing CRISPR-mediated inhibition of two genes involved in BC synthesis, namely *acsAB* and *acsD*, they successfully reduced the BC yield by 15% in the first case and 5% in the second case.

In a similar experiment, another group used CRISPR-mediated inhibition to regulate the UGPase-encoding *galU* gene expression in *K. xylinus* CGMCC 2955. They found that changing the gene’s expression could influence the crystallinity and porosity of the BC pellicle. When repressing this gene, the porosity of BC increased by 0.5-fold. In contrast, the crystallinity of the cellulose network only increased to a certain extent with increasing expression (when the *galU* expression was 30 times higher than in the control group, the crystallinity started to decrease) [144].

The promising opportunities of genetic toolkits extend to BC-composite production where bacteria synthesize another polymer alongside cellulose. Fang et al. [135] demonstrated the biosynthesis of such composites by inserting the curdlan-synthesizing gene (*crdS*) from *Agrobacterium* into *K. xylinus* AY201. The genetically modified bacteria co-produced cellulose and curdlan with minimal changes in the crystallinity of the resulting composite. However, due to the variable pore size, the surface properties of the composite were slightly altered.

To accelerate BC production and make it more cost-effective, researchers came up with the idea of transferring the ability to biosynthesize the cellulose pellicle, which is characteristic of the *Komagataeibacer* genus, to better-studied model organisms with advanced genetic toolkits [13]. Although successful reconstitution of cellulose synthase in *E. coli* was achieved through the heterologous co-expression of genes for proteins BcsA, BcsB, and diguanylate cyclase, the genetically modified bacteria produced amorphous cellulose, highlighting the significance of genes responsible for export and crystallization, namely genes for proteins BcsC and BcsD [134]. Buldum et al. [137] took it further by introducing the entire *bcsABCD* operon into *E. coli*, resulting in the recombinant biosynthesis of cellulose with an exceptional fiber structure measuring from 10 to 20 μm in diameter.

## 4. Conclusions and Perspectives

This review highlights the remarkable potential of BC as an ultrafine nanomaterial comprised of versatile macromolecules with wide-ranging applications. The promising trajectory of BC research offers exciting perspectives for numerous industries and scientific domains. BC’s versatile properties are poised to revolutionize medicine, with potential applications in tissue engineering, wound dressings, and targeted drug-delivery systems. Pharmaceutical advancements may harness BC’s encapsulation capabilities to improve drug stability and bioavailability. Additionally, BC shows great potential in cosmetology, offering opportunities for enhanced skincare products, hair treatments, and wound-healing materials. In electronics and biotechnology, the integration of BC promises the development of flexible and biocompatible materials, facilitating the creation of innovative electronic devices, biosensors, and bioelectronics.

Using synthetic biology tools to genetically engineer BC-producing bacteria introduces a new era of living biomaterials with tailored functionalities, unlocking further possibilities for advanced bioproducts. While these prospects are promising, scaling up BC production remains a challenge that requires continued research to optimize fermentation processes, refine production techniques, and explore sustainable substrates.

By embracing sustainable approaches and employing synthetic biology, BC’s transformative potential can be harnessed across various applications, paving the way for groundbreaking developments in biomaterials and biotechnology. Collaborative efforts across disciplines will be crucial in fully realizing the applications of BC and shaping a more sustainable and innovative future.

## Figures and Tables

**Table 1:** Timeline of key examples intending to improve BC production and modification.

Year	Test Organism	Short Description	Reference
1999	*Acetobacter xylinum* subsp*. sucrofermentans* BPR 2001 (*K. sucrofermentans*)	Introducing the sucrose synthase gene enables sucrose metabolism and improves BC production	[129]
2004	*Acetobacter* ITDI 2.1 (*K. xylinus*)	Introducing the β-galactosidase-encoding gene (*lacZ*) enables lactose metabolism and improves BC production	[130]
2005	*G. xylinus* BPR 2001 (*K. sucrofermentans*)	Knocking out the glucose dehydrogenase-encoding gene (*gdh*) improves BC production	[131]
2006	*A. xylinum* BCRC 12334 (*K. xylinus*)	Expressing the *Vitreoscilla* hemoglobin gene (*vhb*) improves oxygen regulation, cell growth, and BC production	[132]
2006	*G. xylinus* st-60-12 (*K. xylinus*), *L. mali* JCM 1116	Co-culturing *K. xylinus* and *L. mali* improves BC production	[123]
2010	*G. xylinus* ATCC 10245 (*K. xylinus*)	Introducing the UDP-N-acetylglucosamine-synthesizing operon enables the production of BC/chitin copolymer with improved in vivo degradability	[133]
2014	*E. coli* XL1-Blue	Co-expressing cellulose synthase genes for proteins BcsA, BcsB, and diguanylate cyclase (DGC) enables the production of amorphous BC in *E. coli*	[134]
2015	*G. xylinus* AY201 (*K. xylinus*)	Introducing the curdlan-synthesizing gene (*crdS*) enables the production of BC/curdlan composite	[135]
2016	*K. rhaeticus* iGEM	The first standardized genetic toolkit for *K. rhaeticus*, isolated from kombucha tea pellicle, is developed An N-acyl-homoserine lactone (AHL) enables spatial patterning, as shown through red fluorescence (RFP) An RNA-based silencing system repressing the UGPase-encoding gene (*galU*) stops BC production	[136]
2018	*E. coli* GM HMS174 (DE3), *E. coli* GM C41 (DE3)	Introducing cellulose synthase-encoding operon (*bcsABCD*) enables recombinant biosynthesis of BC in *E. coli*	[137]
2019	*K. xylinus* DSM 2325	Overexpressing two carbon metabolism genes (*pgi* and *gnd*) improves BC production	[138]
2019	*K. xylinus* DSM 2325	Introducing the phosphofructokinase-encoding gene (*pfkA*) establishes a glycolytic pathway, increases the level of intracellular ATP, enhances cell growth, and improves BC production	[139]
2019	*K. rhaeticus* iGEM	Co-culturing AHL-inducible and AHL-synthesizing cells enables the developing BC to initiate its red fluorescence	[140]
2019	*G. hansenii* ATCC 53582 (*N. hansenii*), *G. xylinus* ATCC 700178 (*K. xylinus*)*, K. rhaeticus* iGEM	The standardized genetic toolkit is expanded and used in several *Komagataeibacter* species Employing CRISPR-mediated inhibition of two BC biosynthesis genes (*acsAB* and *acsD*) reduces the BC yield	[141]
2019	*K. sucrofermentans*	Adding 6-carboxyfluorescein glucose (6-CF-Glc) to the growth medium enables the production of BC with unnatural green fluorescing capabilities	[122]
2019	*K. hansenii* ATCC 23769 (*N. hansenii*)	Varying the expression of two motility genes (*motA* and *motB*) changes BC morphology	[142]
2019	*Enterobacter* sp. FY-07	Inducing the expression of the colanic acid-encoding operon (*wca*) improves BC water-holding capacity	[143]
2019	*G. hansenii* ATCC 23769 (*N. hansenii*), *E. coli* ATCC 700728	Co-culturing *N. hansenii* and *E. coli* enables the production of BC with improved mechanical properties	[124]
2019	*G. hansenii* ATCC 23769 (*N. hansenii*), *L. lactis* APJ3	Co-culturing *N. hansenii* and *L. lactis* enables the production of BC/hyaluronic acid copolymer	[125]
2019	*A. xylinum* ATCC 23769 (*N. hansenii*), *L. casei*, *L. lactis*, *S. cerevisiae*, *R. palustris*	Co-culturing *N. hansenii* and effective microorganism (EM) improves BC water-holding capacity	[127]
2020	*K. xylinus* CGMCC 2955	Varying the expression of the UGPase-encoding gene (*galU*) changes the crystallinity and porosity of BC	[144]
2021	*K. rhaeticus* iGEM, *S. cerevisiae*	The genetically engineered yeast secretes proteins able to functionalize BC The genetically engineered yeast secretes cellulose-degrading enzymes A yeast-based biosensor system is designed, exhibiting green fluorescence when exposed to β-estradiol (BED)	[63]
2021	*G. xylinus* ATCC 700178 (*K. xylinus*), *R. eutropha* H16 (DSM-428)	Co-culturing *K. xylinus* and *R. eutropha* enables the production of mechanically superior BC/polyhydroxybutyrate nanocomposite	[126]

## Data Availability

Data sharing not applicable.
